# SARS-CoV-2 Variants, South Sudan, January–March 2021

**DOI:** 10.3201/eid2712.211488

**Published:** 2021-12

**Authors:** Daniel Lule Bugembe, My V.T. Phan, Abe G. Abias, James Ayei, Lul Lojok Deng, Richard Lino Loro Lako, John Rumunu, Pontiano Kaleebu, Joseph Francis Wamala, Juma John HM, Dennis Kenyi Lodiongo, Sudhir Bunga, Matthew Cotten

**Affiliations:** Medical Research Council/Uganda Virus Research Institute and London School of Hygiene & Tropical Medicine Uganda Research Unit, Entebbe, Uganda (D.L. Bugembe, M.V.T. Phan, P. Kaleebu, M. Cotten);; National Public Health Laboratory—Ministry of Health, Juba, South Sudan (A.G. Abias, J. Ayei, L.L. Deng, R.L.L. Lako, J. Rumunu);; World Health Organization, Juba (J.F. Wamala, J.J. HM);; US Centers for Disease Control and Prevention, Juba, South Sudan (D.K. Lodiongo, S. Bunga);; University of Glasgow Centre for Virus Research, Glasgow, Scotland, UK (M. Cotten)

**Keywords:** COVID-19, coronavirus disease, SARS-CoV-2, severe acute respiratory syndrome coronavirus 2, viruses, respiratory infections, zoonoses, variants, South Sudan

## Abstract

As the coronavirus pandemic continues, severe acute respiratory syndrome coronavirus 2 (SARS-CoV-2) sequence data are required to inform vaccine efforts. We provide SARS-CoV-2 sequence data from South Sudan and document the dominance of SARS-CoV-2 lineage B.1.525 (Eta variant) during the country's second wave of infection.

As of August 2021, coronavirus disease (COVID-19) had caused >199 million cases and >4.2 million deaths worldwide (*1*). Severe acute respiratory syndrome coronavirus 2 (SARS-CoV-2), the virus that causes COVID-19, is being sequenced to document virus evolution and to inform vaccine efforts. In South Sudan, the COVID-19 index case was confirmed on April 4, 2020 (*2*); it was followed by 2 infection waves, in May–July 2020 and in February–March 2021 (Appendix Figure 1). As of August 3, 2021, a total of 11,063 cases and 119 deaths (*1*) had been reported in South Sudan. An earlier study from South Sudan reported that, after the second wave, 28% of the population showed serologic evidence of infection (*3*).

Chronic underdevelopment caused by prolonged conflicts has left South Sudan with a weak health system and population displacement. Large populations live in camps that may promote rapid spread and amplification of SARS-CoV-2, and poor socioeconomic conditions limit community-based COVID-19 prevention efforts. Monitoring the circulating viral genomic lineages in South Sudan is crucial, especially as vaccination is implemented and novel virus variants appear globally.

## The Study

**Figure 1:**
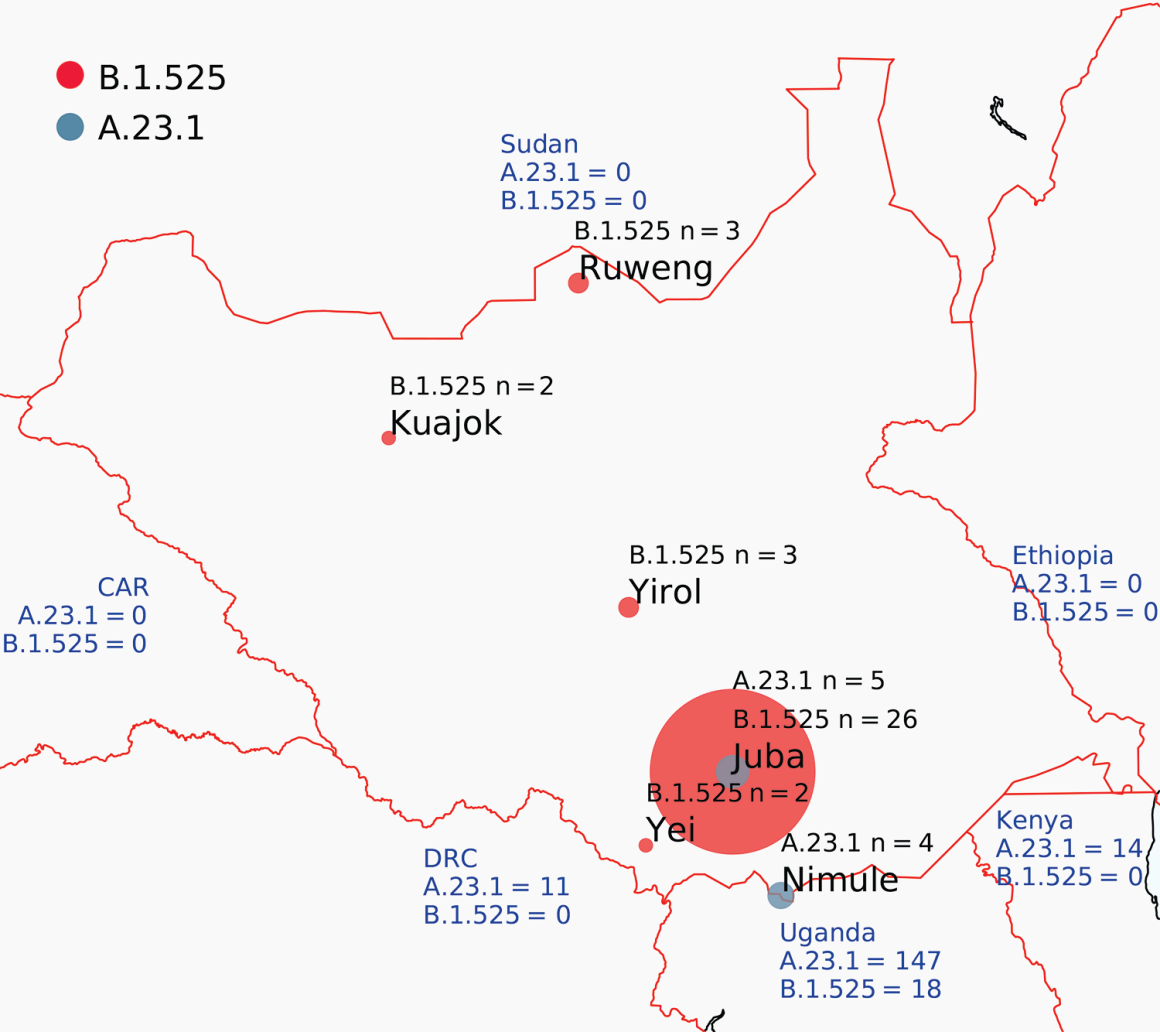
Locations of severe acute respiratory syndrome coronavirus 2 infection case-patients from whom genomes were isolated, South Sudan. Red circles indicate viruses of lineage B.1.525; dark gray circles indicate lineage A.23.1. Circle size is proportional to number of genomes. Blue text shows the number of A.23.1 and B.1.525 genomes reported from neighboring countries. CAR, Central African Republic; DRC, Democratic Republic of the Congo.

As part of South Sudan COVID-19 surveillance, samples were collected from community surveillance, point-of-entry screening, and sentinel site surveillance and tested for SARS-CoV-2 by real-time reverse transcription PCR (RT-PCR) at the National Public Health Laboratory (Juba, South Sudan) (*4*). During the second COVID-19 wave in February–March 2021 (Appendix Figure 1), we tested 56,014 samples for SARS-CoV-2; 6,645 samples tested positive (12% positivity). We selected a set of 70 (1%) of these positive samples for genomic sequencing with these inclusion criteria: diagnostic RT-PCR cycle threshold (C_t_) values <31, from multiple locations (Figure 1), from new arrivals, from death cases, and from sites showing community transmission. We extracted nucleic acid from swab material and generated SARS-CoV-2 genome as previously described (*5*). A total of 45 complete genomes generated from samples collected in January–March 2021 showed a prevalence of 2 lineages: B.1.525 (Eta) and A.23.1 (Figure 1). The A.23.1 lineage, which was observed in October 2020 in Uganda (*6*) and has now spread globally to 26 countries, was one we observed in Juba and Nimule in early January 2021. The A.23.1 case-patients in Nimule, a South Sudan town on the border with Uganda (Figure 1), were travelers returning from Uganda. In Juba, the earliest-reported A23.1 case was in a traveler returning to South Sudan from Uganda. We detected A23.1 for only a short period; from the end of January to the end of March, we detected only B.1.525 genomes (Appendix Table). The B.1.525 lineage, reported earliest in the United Kingdom and Nigeria, has spread to 44 countries and is considered a variant of interest (*7*).

**Figure 2:**
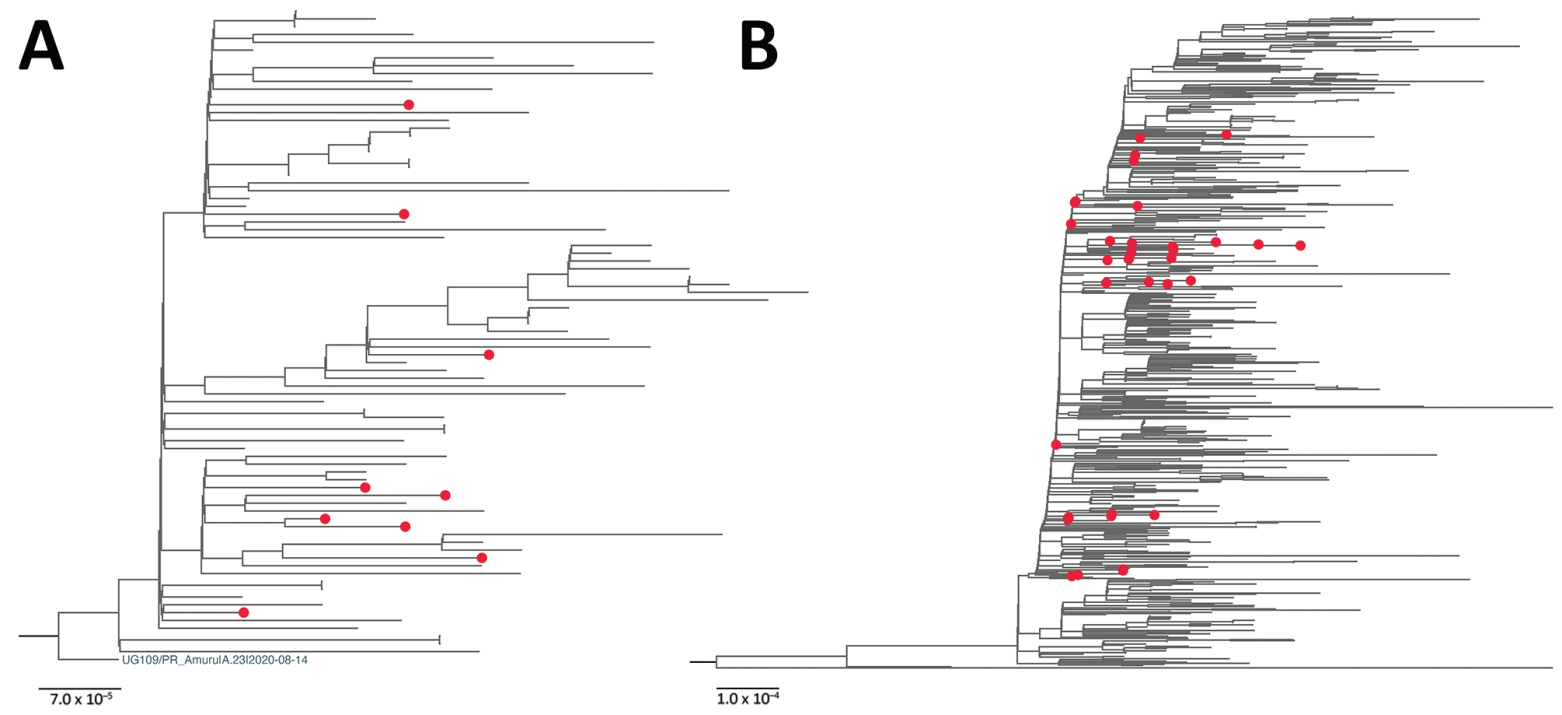
Maximum-likelihood phylogenetic tree of severe acute respiratory syndrome coronavirus 2 viruses from South Sudan (red dots) and reference sequences. A) Lineage A.23.1. All sequences from South Sudan were combined with a subset of all available global A.23.1 genomes, algorithmically thinned. All available global A.23.1 genomes were retrieved from GISAID (https://www.gisaid.org) and aligned, and for the first genome, all genomes closer than 5 hamming distance were removed. This process was continued until the entire set was thinned. This global, thinned A.23.1 set was combined with all South Sudan A.23.1 genomes and used to infer the A.23.1 maximum-likelihood tree. The tree was rooted with the A.23 strain (UG109/PR_Amuru|A.23|2020–08–14). B) Lineage B.1.525. The B.1.525 genome sequences were prepared in the same manner as those for A.23.1 except the hamming distance of 20. Maximum-likelihood phylogenetic trees were constructed in RaxML-NG (*8*) under the general time reversible plus gamma 4 plus invariate sites model as the best-fit model of substitution according to the Akaike information criterion determined by modeltestNG (*9*) and run for 100 pseudoreplicates and visualized using FigTree version 1.4.4 (http://tree.bio.ed.ac.uk/software/figtree). For B.1.525, the tree was midpoint rooted for clarity. Scale bar indicates nucleotide substitutions per site.

Phylogenetic analyses of the South Sudan genomes combined with the available global A.23.1 or B.1.525 genomes were performed to gain insight into the virus movement. The maximum-likelihood trees of both A.23.1 and B.1.525 genome sequences (Figure 2) suggested multiple importations of the strains into South Sudan; South Sudan strains belonged to several sublineages, rather than a single sublineage.

Both A.23.1 and B.1.525 lineages encoded changes in their spike protein (Appendix Figure 2) as well as other parts of the genome and substitutions or deletions in the nonstructural protein 6, open reading frame 3a and 8, and nucleocapsid genes (data not shown), which might be associated with higher transmission or immune evasion. Especially relevant, the A.23.1 genomes encoded spike P681R, which is adjacent to the small (S) 1/S2 furin cleavage site and is also present in the variant of concern B.1.617.2 (Delta) lineage, which is spreading in India and globally and may increase S1/S2 cleavage (*10**,**11*; B. Lubinski et al., unpub. data. http://biorxiv.org/lookup/doi/10.1101/2021.06.30.450632). A related P681H substitution is present in variants of concern B.1.1.7 (Alpha) and P.1 (Beta). The South Sudan B.1.525 genomes encoded a deletion in the N-terminal domain (NTD) at spike positions 69 and 79, which is also present in B.1.1.7 and many other global variants, and a deletion in the spike NTD in positions 141–146, which may help in evasion of host immune responses. The spike D614G substitution may alter the spike protein conformation; the Q677H substitution is near the furin cleavage site and may alter spike processing.

## Conclusions

We describe the patterns of SARS-CoV-2 virus genomics in South Sudan in the second wave of infections during February–March 2021, showing circulation of B.1.525 (Eta) as well as the variant A.23.1. South Sudan faced high transmission of SARS-CoV-2 during this reporting period; our data suggest that the B.1.525 lineage spread widely and progressively increased in frequency in the country during the period. Data from Uganda and Rwanda retrieved from GISAID (https://www.gisaid.org) also showed the appearance of B.1.525 at this time.

A limitation of our study is that sample numbers are low and were limited by the challenges of procurement, shipment, and testing in a harsh and resource-poor environment. Careful sample selection was performed to provide an unbiased description of the epidemic; however, not all positive samples yielded genome sequences. This lack of data could introduce bias in the reported genomes. Nonetheless, the study accurately describes SARS-CoV-2 lineages during the second wave of epidemic in South Sudan.

Substantial land-based traffic with neighboring countries makes it imperative to document the viruses circulating in this region. Careful monitoring of locally circulating viruses as vaccination becomes widespread is essential for interpreting vaccine function and for informing the healthcare systems whether the current vaccines are still a good match for the circulating viruses. We recommend continued genomic surveillance in South Sudan to help with public health responses, especially as new waves of infections come to the country and continent.

Appendix Additional information about severe acute respiratory syndrome coronavirus 2 virus variants in South Sudan.
