# Supplementary material for: SARS-CoV-2 Variants, South Sudan, January–March 2021
Source: Emerg Infect Dis. 2021 Dec;27(12):3133–6. doi: 10.3201/eid2712.211488 (PMC8632182; doi:10.3201/eid2712.211488)
Supplement: Appendix — Additional information about severe acute respiratory syndrome coronavirus 2 virus variants in South Sudan. [file 21-1488-Techapp-s1.pdf]

Article DOI: https://doi.org/10.3201/eid2712.211488

SARS-CoV-2 Variants, South Sudan, January–March 2021

Appendix

Appendix Table. Summary of samples and resulting genomes from study of SARS-CoV-2 variants, South Sudan, January–March 2021.

Genome	Lineage	C_t	Category	Patient outcome	Local address	Sample date
1	A.23.1	26.3	Screening_4Travel	Survived	Nimule	2021 Jan 8
2	A.23.1	24.6	Screening_4Travel	Survived	Nimule	2021 Jan 8
3	A.23.1	23.9	Screening_4Travel	Survived	Nimule	2021 Jan 13
4	A.23.1	NA	NA	NA	Juba	NA
5	A.23.1	26.34	Screening_4Travel	Survived	Nimule	2021 Jan 18
6	A.23.1	17.3	Screening_4Travel	Survived	Juba	2021 Jan 20
7	A.23.1	28	Screening_4Travel	Survived	Juba	2021 Jan 25
8	A.23.1	28.67	Screening_4Travel	Survived	Juba	2021 Jan 26
9	A.23.1	26.3	Screening_4Travel	Survived	Juba	2021 Jan 27
10	B.1.525	23.2	asymptomatic follow up	Survived	Juba	2021 Jan 23
11	B.1.525	23	Screening_4Travel	Survived	Juba	2021 Jan 23
12	B.1.525	22.83	Screening_4Travel	Survived	Juba	2021 Jan 26
13	B.1.525	24.56	Alert/susp_WithSymp	Survived	Yirol	2021 Jan 27
14	B.1.525	27.3	Screening_4Travel	Survived	Juba	2021 Jan 28
15	B.1.525	27.7	Screening_4Travel	Survived	Juba	2021 Jan 28
16	B.1.525	26.1	Screening_4Travel	Survived	Juba	2021 Jan 28
17	B.1.525	21.48	Alert/susp_WithSymp	Survived	Yirol	2021 Jan 28
18	B.1.525	16.3	Alert/susp_WithSymp	Survived	Juba	2021 Feb 5
19	B.1.525	17	Alert/susp_WithSymp	Survived	Juba	2021 Feb 6
20	B.1.525	18.49	Alert/susp_WithSymp	Survived	Yirol	2021 Feb 9
21	B.1.525	23.01	Screening_4Travel	Survived	Kuajok	2021 Mar 2
22	B.1.525	27.56	Screening_4Travel	Survived	Kuajok	2021 Mar 2
23	B.1.525	24.25	Screening_4Travel	Survived	Juba	2021 Mar 2
24	B.1.525	29.19	Alert/susp_WithSymp	Survived	Juba	2021 Mar 3
25	B.1.525	27.48	Screening_4Travel	Survived	Juba	2021 Mar 3
26	B.1.525	20.27	Cont_Without sympt	Survived	Juba	2021 Mar 4
27	B.1.525	24.34	Screening_4Travel	Survived	Juba	2021 Mar 4
28	B.1.525	23.8	Alert/susp_WithSymp	Survived	Juba	2021 Mar 4
29	B.1.525	22.02	Alert/susp_WithSymp	Survived	Juba	2021 Mar 5
30	B.1.525	22.49	Alert/susp_WithSymp	Survived	Ruweng	2021 Mar 5
31	B.1.525	19.33	Screening_4Travel	Survived	Gudele	2021 Mar 7
32	B.1.525	21.17	Screening_4Travel	Survived	Juba	2021 Mar 8
33	B.1.525	27.44	Screening_4Travel	Survived	Juba	2021 Mar 8
34	B.1.525	26.71	Screening_4Travel	Survived	Juba	2021 Mar 8
35	B.1.525	21.52	Screening_4Travel	Survived	Atlabara	2021 Mar 9
36	B.1.525	22.6	Alert/susp_WithSymp	Survived	Ruweng	2021 Mar 9
37	B.1.525	24.99	Screening_4Travel	Survived	Juba	2021 Mar 10
38	B.1.525	30.59	Alert/susp_WithSymp	Survived	Yei	2021 Mar 11
39	B.1.525	22.41	Alert/susp_WithSymp	Survived	Ruweng	2021 Mar 11
40	B.1.525	25.77	Postmortem_withsymp	Died	Yei	2021 Mar 12
41	B.1.525	25.76	Screening_4Travel	Survived	Juba	2021 Mar 15
42	B.1.525	15.81	Screening_4Travel	Survived	Juba	2021 Mar 15
43	B.1.525	20.98	Cont_Without sympt	Survived	Juba	2021 Mar 18
44	B.1.525	26.9	Alert/susp_WithSymp	Survived	Juba	2021 Mar 18
45	B.1.525	23.82	Alert/susp_WithSymp	Survived	Juba	2021 Mar 20

*Complete genomes were lineage typed using the Pango tool (*1*). NA, data not available.

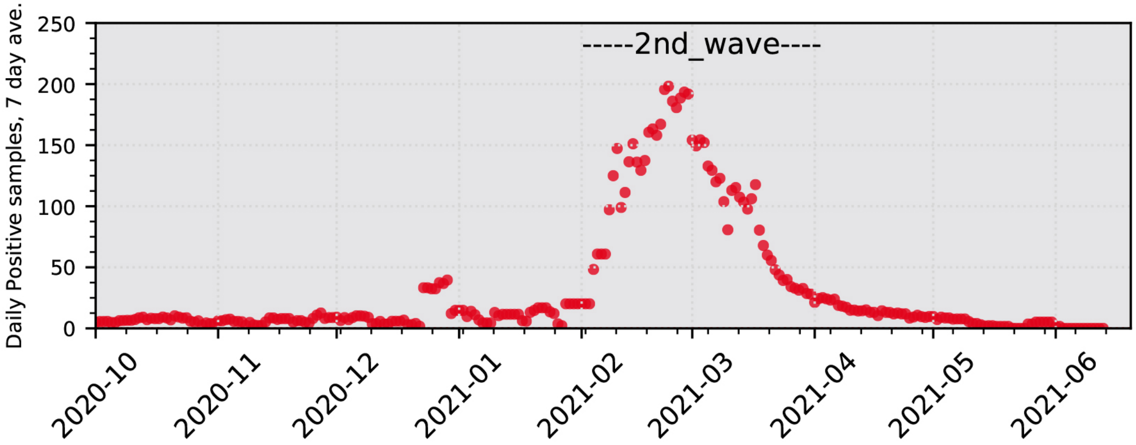

Appendix Figure 1. Reported severe acute respiratory syndrome coronavirus 2 infections in South Sudan, shown as the 7-day average of daily reported SARS-CoV-2 infections (*2*).

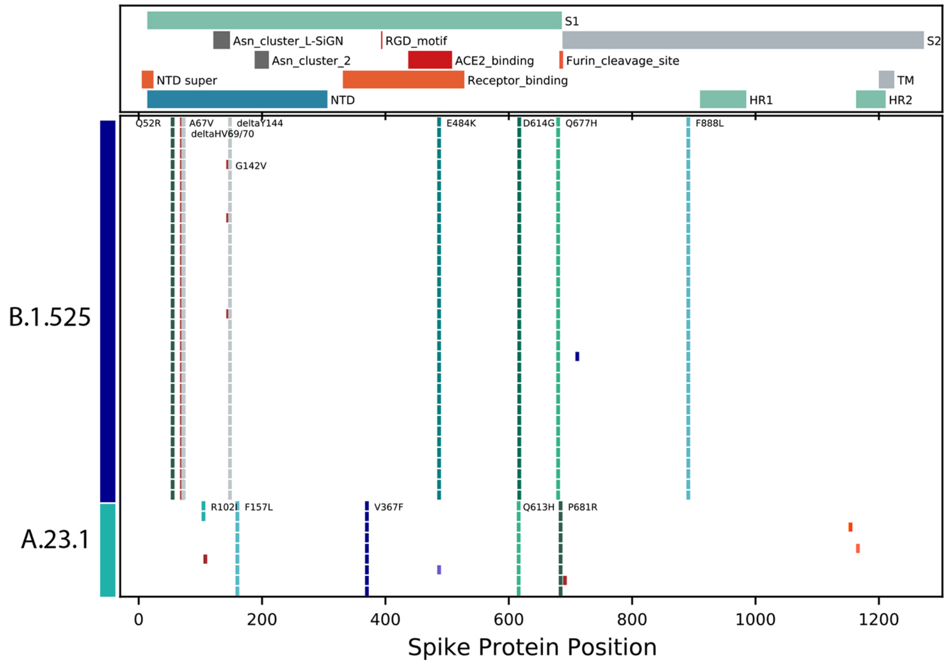

Appendix Figure 2. Changes in the spike protein compared to the NC_045512 reference strain of severe acute respiratory syndrome coronavirus. Each line represents the encoded spike protein sequence from a single genome; sequences are ordered by lineage and then by sample collection date. Markers indicate the positions of amino acid (aa) differences from the reference strain; only changes observed in multiple genomes were annotated with the annotation (original aa position new aa) located either to the left or right of the most recent instance of the substitution. Important spike protein features are indicated above the graph. HR1, helical repeat 1; HR2, helical repeat 2; NTD, N terminal domain; NTD super, N terminal domain supersite; RBD, receptor-binding domain; S1, spike 1; S2: Spike 2; TM, transmembrane domain.

References

1. O'Toole Á, Hill V, McCrone JT, Scher E, Rambaut A. Pangolin COVID-19 lineage assigner. 2020 [cited 2021 Sep 3]. https://pangolin.cog-uk.io/

2. Center for Systems Science and Engineering (CSSE) at Johns Hopkins University. COVID-19 dashboard. 2020 [cited 2021 Jan 24]. https://www.arcgis.com/apps/opsdashboard/index.html#/bda7594740fd40299423467b48e9ecf6
